# 'The intraabdominal bleeding with an inguinal defect that mimicking a femoral vein aneurysm'. A case report

**DOI:** 10.1186/1755-7682-4-13

**Published:** 2011-04-17

**Authors:** Bülent Kaya, Orhan Bat, Nuriye Esenbulut, Kemal Memisoglu

**Affiliations:** 1 Fatih Sultan Mehmet Training and Research Hospital, Department of General Surgery Istanbul-Turkey

## Abstract

Inguinal hernia repair is commonly performed operation in surgical practice. Thirty-five years old female patient was operated with the diagnosis of inguinal hernia. The inguinal defect was misdiagnosed as femoral vein aneurysm in surgical exploration. The postoperative diagnostic imaging revealed that the inguinal defect had been confused as femoral vein aneurysm due to intra-abdominal hemorrhogical fluid after an ovarian cyst rupture.

## Background

Inguinal hernia repair is one of the most commonly performed operation in surgical practice. Sometimes, challenging cases may be encountered. An inguinal hernia can mimic many pathologies and become a surgical dilemma, even for the skilled surgeon [1]. The correct diagnosis of an inguinal mass is important in order to avoid performing non-therapeutic surgery and serious complications.

We reported a patient who had been explorated with the diagnosis of inguinal hernia. The defect was diagnosed as femoral vein aneurysm in surgical exploration. The postoperative diagnostic imaging revealed that the inguinal defect had been confused as femoral vein aneurysm due to intra-abdominal hemorrhogical fluid after an ovarian cyst rupture. We concluded that an inguinal mass can be a diagnostic dilemma with treatment problems.

## Case Presentation

Thirty-five years old female patient presented with pain and swelling in her left inguinal region. She was very thin in appreance. On physical examination, an inguinal mass, bulging with coughing about 3 × 4 cm in diameter was palpated. She had been operated for left ovarian cyst rupture about 8 years ago. She was diagnosed as inguinal hernia and prepared for elective inguinal hernia repair. The routine preoperative laboratory tests were within normal limits.

**Figure 1:**
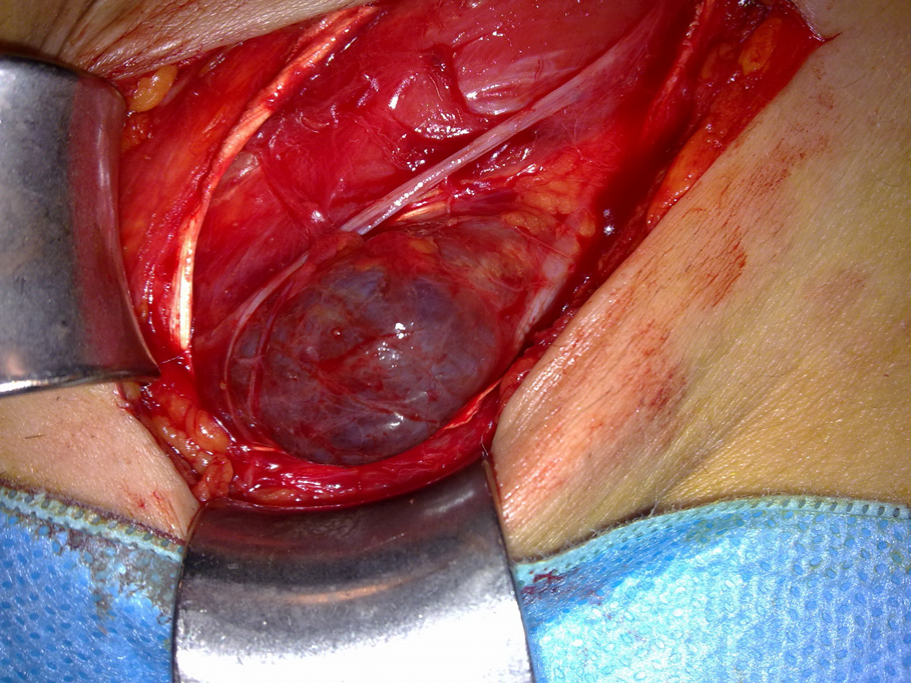
**Inguinal image that mimics a venous aneurysm**.

The inguinal canal was explorated in operation. A lump, bluish-purple in color, about 3 × 4 cm in diameter was detected (Figure 1). It was emerging from the inferior part of the inguinal canal. It was accapted as a vascular lesion with it's appreance. The cardiovascular surgeon was invited for consultation. The dissection was performed up to the conjoint tendon and iliopubic tract. It was decided that the lesion was a femoral vein aneurysm and elective surgery should be recommended.

**Figure 2:**
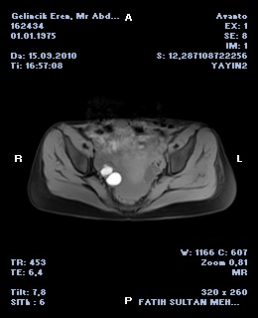
**Hemorrhagical fluid was detected in MRI**.

**Figure 3:**
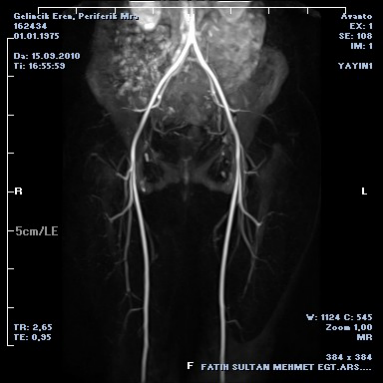
**Peripheral MR Angiography was normal**.

We performed pelvic ultrasonography (USG) and pelvic Magnetic Resonance Imaging (MRI) postoperatively. Two ovarian cysts about 2.5 × 3.5 cm and 3 × 2.5 cm in diameter were detected in left ovary. There was also free hemorrhogic fluid in lower abdominal quadrants (Figure 2). The tumor markers including AFP, CEA, CA 125, CA 15-3 and CA 19-9 were within normal limits.The color doppler USG of inguino-femoral region was normal. There were no femoral arterial aneurysm in MRI angiography (Figure 3). The intra-abdominal hemorrhogical fluid had been mimiced the venous aneurysm. The patient was decided to followed-up and elective hernia repair was recommended.

Many surgical pathologies may present as inguinal swelling such as hernia, lymphadenopathy, varices, and endometriosis [2,3] Sometimes it is difficult to diagnose an inguinal mass definitively even in surgical exploration. Preoperative or intraoperative correct diagnosis is mandatory for performing a proper management.

A venous aneurysm is usually defined as a venous dilation which comminicates with a main venous structure by a channel. It may cause serious complications such as deep vein thrombosis and pulmonary emboli [4]. Venous aneurysms can be seen in every major veins and may be misdiagnosed as an inguinal hernia [5]. Femoral vein aneurysm is a rare clinical pathology [6]. It may also be misdiagnosed as an inguinal or femoral hernia. Castaldo ET, have been presented a femoral vein aneurysm simulating an inguinal hernia and added that only five cases have been reported in literature before their case report [7]. On the other hand, an inguinal hernia, mimicking a venous aneurysm is even more rare clinical condition. To our knowledge, there is no such a case that was reported in English literature up to date. Our patient was presented with classical findings of inguinal hernia. She had a bulging inguinal mass enlarging with increase in intra-abdominal pressure. The appreance of lamp was confusing at operation. After exploration with blunt dissections, the lesion was diagnosed as femoral vein aneurysm intra-operatively. The surgical intervention was terminated. An elective aneurysm repair after diagnostic evaluation was planned.

The postoperative diagnostic evaluation was performed. The arterial and venous system was imaged with MRI angiography and color doppler ultrasonography. There were no aneurysm in both femoral artery or vein. The only detected pathology was ovarian cysts with intra-abdominal hemorrhogic fluid in pelvic MRI. Marcucci G recommended doppler USG for venous aneurysms in inguinal region and lower extremities [8]. The bulging lesion that had been diagnosed as femoral vein aneurysm was a peritoneal sac (inguinal defect) filled with hemorrhogical fluid.

## Conclusion

It is also important to conclude that although it is very rare, an inguinal defect with an intra abdominal bleeding can mimic a vascular lesion like venous aneurysm. The definitive diagnosis of inguinal mass is mandatory for proper treatment. Radiological evaluation of inguinal mass before surgery can gain clinical importance.

## Competing interests

The authors declare that they have no competing interests.

## Authors' contributions

BK, OB, NE, KM were involved in the direct care of the patient. In addition, BK was responsible for drafting the manuscript and OB, NE and KM helped to draft the manuscript. All authors have read and approved the final manuscript.

## Consent

Written informed consent was obtained from the patients for publication of this case report and accompanying images. A copy of the written consents is available for review by the Editor-in-Chief of this journal.

## References

[B1] Rutkow IM Robbins AW Demographic, classificatory, and socioeconomicaspects of hernia repair in the United States Surg Clin North Am 1993 73 413 426 8497793 10.1016/s0039-6109(16)46027-5

[B2] Chi C Taylor A Munjuluri N Abdul-Kadir R A diagnostic dilemma: round ligament varicosities in pregnancy Acta Obstet Gynecol Scand 2005 84 1126 1127 10.1111/j.0001-6349.2005.00120c.x 16232186

[B3] Oh SN Jung SE Rha SE Lim GY Ku YM Byun JY Lee JM Sonography of various cystic masses of the female groin J Ultrasound Med 2007 26 1735 1742 18029925 10.7863/jum.2007.26.12.1735

[B4] Gillespie DL Villavicencio JL Gallagher C Chang A Hamelink JK Fiala LA O'Donnell SD Jackson MR Pikoulis E Rich NM Presentation and management of venous aneurysms J Vasc Surg 1997 26 845 852 10.1016/S0741-5214(97)70099-5 9372824

[B5] Majeski J Surgical repair of primary saphenous vein aneurysm of the proximal leg after initial presentation as an inguinal hernia Am Surg 2002 68 999 1002 12455795

[B6] Pandey V Wolfe JH Superficial femoral vein aneurysm with massive pulmonary embolism J R Soc Med 2003 96 460 1 10.1258/jrsm.96.9.460 12949206 PMC539605

[B7] Castaldo ET Williams EH Dattilo J Passman M Naslund T Guzman RJ Common femoral vein aneurysm simulating an inguinal hernia Am Surg 2005 71 591 4 16089125

[B8] Marcucci G Accrocca F Antignani PL Siani A An isolated aneurysm of the thigh anterolateral branch of the greater saphenous vein in a young patient presenting as an inguinal hernia Interact Cardiovasc Thorac Surg 2010 10 654 5 10.1510/icvts.2009.230318 20067986

